# Sp1/Sp3 and DNA-methylation contribute to basal transcriptional activation of human podoplanin in MG63 versus Saos-2 osteoblastic cells

**DOI:** 10.1186/1471-2199-8-20

**Published:** 2007-03-07

**Authors:** Brigitte Hantusch, Romana Kalt, Sigurd Krieger, Christina Puri, Dontscho Kerjaschki

**Affiliations:** 1 Institute of Clinical Pathology, Medical University of Vienna, Währinger Gürtel 18-20, A-1090 Vienna, Austria

## Abstract

**Background:**

Podoplanin is a membrane mucin that, among a series of tissues, is expressed on late osteoblasts and osteocytes. Since recent findings have focussed on podoplanin's potential role as a tumour progression factor, we aimed at identifying regulatory elements conferring *PDPN *promoter activity. Here, we characterized the molecular mechanism controlling basal *PDPN *transcription in human osteoblast-like MG63 versus Saos-2 cells.

**Results:**

We cloned and sequenced 2056 nucleotides from the 5'-flanking region of the *PDPN *gene and a computational search revealed that the TATA and CAAT box-lacking promoter possesses features of a growth-related gene, such as a GC-rich 5' region and the presence of multiple putative Sp1, AP-4 and NF-1 sites. Reporter gene assays demonstrated a functional promoter in MG63 cells exhibiting 30-fold more activity than in Saos-2 cells. *In vitro *DNase I footprinting revealed eight protected regions flanked by DNaseI hypersensitive sites within the region bp -728 to -39 present in MG63, but not in Saos-2 cells. Among these regions, mutation and supershift electrophoretic mobility shift assays (EMSA) identified four Sp1/Sp3 binding sites and two binding sites for yet unknown transcription factors. Deletion studies demonstrated the functional importance of two Sp1/Sp3 sites for *PDPN *promoter activity. Overexpression of Sp1 and Sp3 independently increased the stimulatory effect of the promoter and podoplanin mRNA levels in MG63 and Saos-2 cells. In SL2 cells, Sp3 functioned as a repressor, while Sp1 and Sp3 acted positively synergistic. Weak *PDPN *promoter activity of Saos-2 cells correlated with low Sp1/Sp3 nuclear levels, which was confirmed by Sp1/Sp3 chromatin immunoprecipitations *in vivo*. Moreover, methylation-sensitive Southern blot analyses and bisulfite sequencing detected strong methylation of CpG sites upstream of bp -464 in MG63 cells, but hypomethylation of these sites in Saos-2 cells. Concomitantly, treatment with the DNA methyltransferase inhibitor 5-azaCdR in combination with trichostatin A (TSA) downregulated podoplanin mRNA levels in MG63 cells, and region-specific *in vitro *methylation of the distal promoter suggested that DNA methylation rather enhanced than hindered *PDPN *transcription in both cell types.

**Conclusion:**

These data establish that in human osteoblast-like MG63 cells, Sp1 and Sp3 stimulate basal *PDPN *transcription in a concerted, yet independent manner, whereas Saos-2 cells lack sufficient nuclear Sp protein amounts for transcriptional activation. Moreover, a highly methylated chromatin conformation of the distal promoter region confers cell-type specific podoplanin upregulation versus Saos-2 cells.

## Background

Podoplanin represents a type I integral membrane protein of the mucin-type family. Its 162 aa protein core is extensively O-glycosylated, which results in a doubling of the molecular weight up to 45 kDa. The podoplanin mRNA was identified for the first time in the murine osteoblastic cell line MC3T3-E1 [1], and later it was found to be expressed in murine colon carcinoma and melanoma cells [2], in the stromal cells of peripheral lymphoid tissue [3], in skeletal muscle, heart, placenta, lung, skin, bone and brain [4,5]. Podoplanin has also been detected in the ciliary epithelium of the eye [6], in transformed epidermal keratinocytes and fibroblasts [7] and in the choroid plexus and neuronal cells of the CNS [8]. In our lab, podoplanin has been discovered as a membrane protein of glomerular epithelial cells, which was found to be downregulated in puromycin nephrosis and caused a flattening of podocytes, hence its designation "podoplanin" [9]. Moreover, podoplanin has been found in endothelia of lymphatic vessels [4] and has been established as a discrimination marker between lymphatic (LEC's) and blood endothelial cells (BEC's) [10]. The widespread localization of podoplanin has lead to a multiplicity of synonyms for its expression forms of human, murine and rat origin: murine OTS-8 [1], gp38 [3], T1α [11], PA2.26 antigen [7], RANDAM-2 [8] or Aggrus [12], rat E11 antigen [4] or RTI40 [13], and human gp36 [5].

The exact biological function of podoplanin remains controversial and seems to be versatile. Though its highly abundant expression in a multiplicity of tissues, distinct cell-specificities and functions have been described. In the lung, podoplanin has been shown to be restricted to type I lung alveolar cells [11], where it also functions as the main receptor for influenza C virus [14]. Also quite early, podoplanin has been described as a platelet aggregating factor in murine and human colon carcinoma [15] and murine melanoma [2]. Podoplanin/T1α deficiency in mice lead to disruption of normal lymphatic vasculature formation and development of lymphedema [16]. Moreover, podoplanin has been demonstrated to bind chemokines in the lymphatic vessels of renal transplants [17] and to promote cell motility by downregulating E-cadherin expression in oral squamous cell carcinomas [18]. Recent studies, therefore, have focussed on its potential role as a key player in tumour vessel formation [19] and as a tumour progression factor in skin [20] and germ line carcinomas [21]. Podoplanin becomes upregulated in cells of the invasive cancer front, where it induces filopodia formation and promotes tumour cell migration [22].

Rat podoplanin/E11 has been reported to be a marker for late steps in the differentiation pathway of the osteoblastic lineage, being expressed by mature osteoblasts and newly formed osteocytes [4]. The human osteosarcoma cell lines MG63 and Saos-2 used in this study represent differentiated late stage osteoblasts, nonetheless showing different degrees of maturation. Whereas MG63 cells exhibit a premature fibroblast-like state, Saos-2 cells display an epithelial phenotype and appear more rounded [23]. Podoplanin/E11 was detected in a restricted subset of osteoblastic cells which is in direct contact with bone matrix, suggesting a functional role as an anti-adherent molecule according to its high sialic acid content. Nevertheless, the function of podoplanin in the bone is unknown.

Despite considerable progress that has been made in describing the expression pattern of podoplanin under normal and various pathological conditions, little is known about the mechanisms leading to the widespread gene activity of podoplanin. Due to its certain specificities and potential role in tumour progression, the regulation of *PDPN *gene expression is of high interest. In an early study, podoplanin/OTS-8 could be induced by phorbolester in the mouse osteoblastic cells MC3T3-E1 [1], and therefore it was defined as a target gene for early response proteins. However, the molecular mechanism underlying podoplanin expression in osteoblasts remains largely unclear. In rat alveolar type I cells, Sp1 elements and a thyroid transcription (TGT3/HNF-3) factor site have been identified as essential for transcriptional activation of podoplanin/T1α [24,25]. In lymphatic endothelial cells, podoplanin/T1α is an early responder to the lymphatic-specific master regulator Prox-1 [26], and recently, podoplanin expression was shown to be under control of endogenously produced IL-3 in human lymphatic endothelial precursor cells [27]. However, no direct cis- and trans-acting regulatory elements involved in cell-specific upregulation of human podoplanin have been elucidated so far.

In this study, we demonstrate a cell-specific difference between MG63 and Saos-2 cells in the binding of nuclear proteins to sequences immediately adjacent and more distal to the transcription start site of the *PDPN *promoter. We identify four Sp1/Sp3 binding-sites inside a 654 bp element upstream of the *PDPN *gene and establish that two of them mediate basal transcription in MG63 cells, whereas Saos-2 cells seem to lack sufficient amounts of nuclear Sp1/Sp3 for *PDPN *promoter activation. We show that these two functional Sp-binding sites may involve further activator elements. Moreover, we assess the functional hierarchy of another regulatory system, the chromatin modification, which is strongly altered in MG63 versus Saos-2 cells distal of the core promoter and leads to enhanced expression of podoplanin.

## Results

### Podoplanin mRNA expression in the human osteoblastic cell lines MG63 and Saos-2

**Figure 1:**
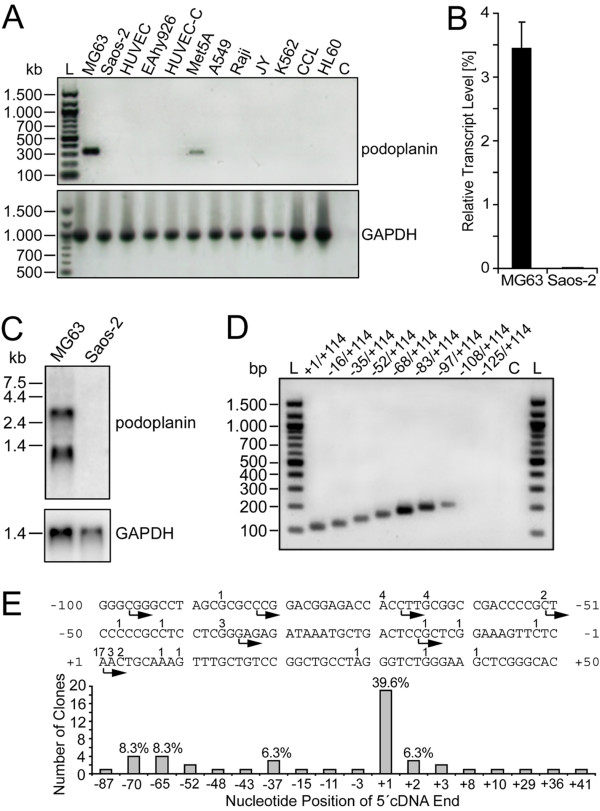
**Podoplanin mRNA transcription and transcriptional initiation in the human osteoblast-like cell line MG63**. A) *PDPN *transcription was analyzed using primers specific for podoplanin cDNA (see "Material and Methods"). The human cell lines are: MG63 and Saos-2, osteosarcoma cells; HUVEC, umbilical vein endothelial cells; EAhy926, endothelial-like cells; Met5A, pleural mesothelial cells; A549, type II alveolar lung carcinoma cells; Raji, Burkitt's B-lymphoma cells; JY, B-lymphoblastoid cells; K562, erythroleukemia cells; CCL, "centrocyte-like" B-lymphocytes; HL60, promyeloid cells. B) Real-time PCR analysis of *PDPN *transcription in MG63 versus Saos-2 cells. Results ± S.D. of three independent PCRs are shown. Transcript levels are relative to GAPDH mRNA content. C) Northern blot analysis of podoplanin mRNA fragments in MG63 versus Saos-2 cells. D) RT-PCR determination of transcription initiation sites in MG63 cells. The lane numbers correspond to the RT-PCR 5' primers in Table 1. They were tested together with 3' primer hPPrev (+114/+96) for their ability to amplify the corresponding sequences in cDNA preparations of MG63 cells. PCR products are shown on an agarose gel stained with ethidium bromide. Lanes L: DNA ladder, Lane C: Negative control containing no template DNA for PCR reaction. E) Sequencing results of 48 clones retrieved by 5' RACE from MG63 cells. The 5' cDNA endings and their frequency are indicated above the sequence stretch.

To identify cell lines which are positive for podoplanin expression, reverse transcription PCRs were performed with a series of human cell lines of different origin (Fig. 1A). The osteoblastic osteosarcoma cell line MG63 exhibited a strong RT-PCR signal, whereas Saos-2 osteosarcoma cells lacked it. This result was confirmed by real-time PCR analyses showing that MG63 cells contained three logs more podoplanin mRNA transcripts than Saos-2 cells. While in MG63 cells the podoplanin mRNA population represented approximately 3% of GAPDH mRNA population, it made up just 0.003% in Saos-2 cells (Fig. 1B). A Northern blot analysis revealed the presence of two podoplanin transcript variants in MG63 cells (Fig. 1C). A 2.8 kb transcript corresponding to the full length mRNA was seen, and a shorter species was detected at 1.1 kb. As determined by 3' RACE analysis using MG63-derived cDNA, this short mRNA species represented a transcriptional variant ending after a cryptic polyA-signal at bp 345 of the 2068 bp 3'-UTR (data not shown).

**Table 1:** Primer sequences used in this study.

Name	Sequence (5'→3') ^a^	Experiment ^b^
hPP rev (+207/+178)	CACATCGTTCCCGTTGAGTTGTTGCTCTCC	RT-PCR
hPP 1 (+1/+19)	AACTGCAAAGTTTGCTGTC	RT-PCR
hPP 2 (-16/+3)	CGCTCGGAAAGTTCTCAAC	RT-PCR
hPP 3 (-35/-17)	GAGAGATAAATGCTGACTC	RT-PCR
hPP 4 (-52/-33)	CTCCCCCGCCTCCTCGGGA	RT-PCR
hPP 5 (-68/-50)	CTTGCGGCCGACCCCGCTC	RT-PCR
hPP 6 (-83/-65)	CCGGACGGAGACCACCTTG	RT-PCR
hPP 7 (-97/-79)	CGGGCCTAGCGCGCCCGGA	RT-PCR
hPP 8 (-108/-90)	GAGCCCGAGGGCGGGCCTA	RT-PCR
hPP 9 (-125/-107)	TTGGCGCCGGCCAAACAGA	RT-PCR
hPP rev (+114/+96)	AGCCTGGAGGAGCGCGAC	RT-PCR
FII (-1885/-1866)	GCAATGAGCTCATCCACTAACTCCTTCATCC	Cloning, LA
F I (-857/-840)	GCAATGAGCTCACGTAAGTTCTCTGGTTG	Cloning, LA
RII (-783/-803)	GCAATCTCGAGAATCCTCACTTTACTGGTTTA	Cloning
RI (+154/+171)	GCAATCTCGAGCCGAGCAGCAAGATTCTG	Cloning, LA
F1 (-1642/-1616)	TTGAATAAAGCGGGCCATC	LA
F2 (-1378/-1360)	TCATCTTCTAGGTTCAGGT	LA
F3 (-1104/-1086)	ACAATCTCTCCTGTGATTC	LA
F4 (-666/-658)	ACAGCATCGCAGCGCTGGA	LA
F5 (-498/480)	ACCAGCGCGGGCCGCCTCT	LA
F6 (-361/-343)	CACTTCTGCCCATAACAAG	LA
F7 (-261/-243)	AACCCCGTTTGTTGCATGT	LA
F8 (-232/-214)	CCTCCTGTTGTTGAGCAGA	LA
F9 (-186/-168)	AGTCCTGGCGGCCCCCGCA	LA
F10 (-159/-141)	CCTGTAACTTTAAACCTGG	LA
F11 (-114/-96)	CAAACAGAGCCCGAGGGCG	LA
F12 (-76/-58)	GAGACCACCTTGCGGCCGA	LA
F13 (-38/-20)	CGGGAGAGATAAATGCTGA	LA
F14 (-26/-8)	ATGCTGACTCCGCTCGGAA	LA
fp 1	CCGCTCCCCCGCCTCCTCGGGA	EMSA
fp 1 mut	CCGCTCC***A***C***ATAA***TCCTCGGGA	Mutation
fp 2	GAGCCCGAGGGCGGGCCTAGCG	EMSA
fp 2 mut	GAGCCCGA***T***G***ATAA***GCCTAGCG	Mutation
fp 3	GATCATCGTTTTGGCGCCGGCCAA	EMSA
fp 4	CAGATGACGGCCAACTTTTTTTGTTTTTAAGCCATCAAA	EMSA
fp 5	CCAAACTCCCCCCACCAGCCAG	EMSA
fp 5 mut	CCAAACTC***TA***C***AG***ACCAGCCAG	Mutation
fp 6	TTTTCAAAACTGCCAAAGCC	EMSA
fp 7	CACCGCTTGGCCCGGCCTAATCC	EMSA
fp 7 mut	CACCGCTTGG***AT***C***TTG***CCTAATCC	Mutation
fp 8	GGACACAGGTTTGCGACCAGGACGGT	EMSA
Bis-fwd 1	GTTAGGGTTTGTAGTTTTGGTGG	BS
Bis-rev 1	CACCACAACCACAAACCCAACCT	BS
Bis-fwd 2	GAGGGTGAATTAGGTTTGGAAGGGG	BS
Bis-rev 2	CAACCCACACTAATCAATTACACCC	BS
Bis-fwd 3	GAGTTGTTGGTTTTATTTGGTTTAGGAGGGTGAG	BS
Bis-rev 3	CTATTCTACTTATATATAACTCCAAAATCACACAC	BS

^a ^Sequences protected in DNase I footprinting are underlined; mutated nucleotides are indicated in bold italics.

^b ^RT-PCR, reverse transcription PCR; LA, Luciferase assay; EMSA, electrophoretic mobility shift assay; BS, bisulfite sequencing.

**Figure 2:**
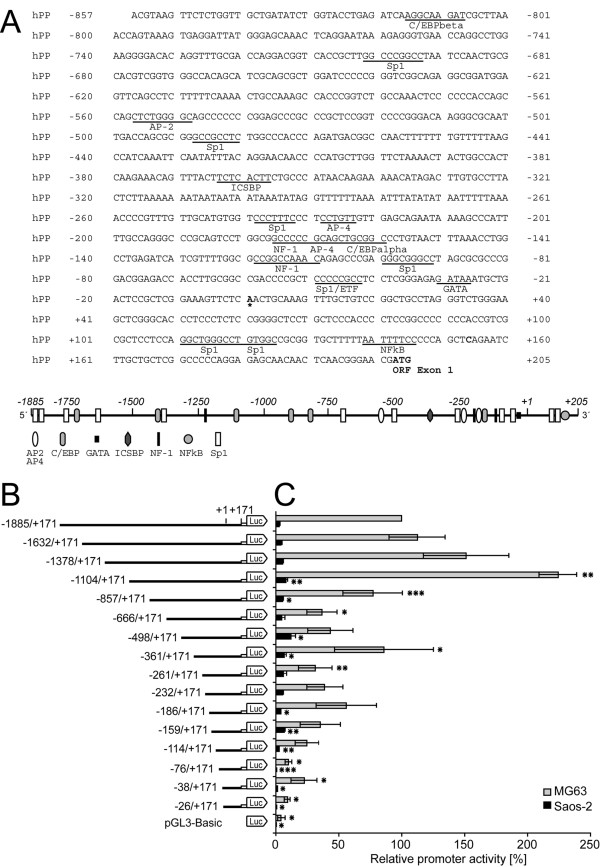
**Structure and activity of the human *PDPN *5'-flanking region in MG-63 and Saos-2 cells**. A) Nucleotide sequence and putative regulatory elements within bp -857/+205 of the 5'-flanking region of the human *PDPN *gene. The major transcription start site is indicated with an asterisk at +1. Putative regulatory elements highlighted by underlining were identified using the Transfac database. Shown are only sites which are conserved between the human, murine and rat promoter. Numbers on the left and right refer to the nucleotide position relative to the major transcription start site. The scheme below shows the relative positions of these putative transcription factor binding sites within bp -1885/+205. AP, activating enhancer binding protein; C/EBPβ, CCAAT/enhancer binding protein β; ETF, EGFR-specific transcription factor; ICSBP, interferon consensus sequence binding protein; NF-1, nuclear factor 1. B) Schematic representation of stepwise 5' deletions of the *PDPN *5' flanking region cloned upstream of the luciferase reporter gene into the pGL3 vector. Negative numbers indicate the 5' end of the promoter fragment relative to the major transcription start site at +1. C) Luciferase activity of 5' deletion constructs were assessed in MG63 and Saos-2 cells 24 h after transfection with equal stoichiometric plasmid amounts and a constant amount of internal control *Renilla *luciferase plasmid as described under "Methods". Luciferase activity was normalized to *Renilla *activity and is relative to the longest bp -1885/+171 construct, which was set at 100%. The average ± S.D. for three to six independent transfection experiments, each performed in triplicates, is shown. Statistical analysis was performed using student's *t*-test, and is as follows: *, p < 0.05, **, p < 0.01, ***, p < 0.001. The statistical analysis indicates a significant promoter activity difference between the consecutive deletion-constructs.

Reverse transcription PCR applying a series of nine ascending 5' primers suggested that in MG63 cells, podoplanin mRNA was initiated at multiple sites mostly located between bp -97 and +1 of the 5'-flanking region (Fig. 1D). In 5' RACE experiments using MG63-derived cDNA, the 5'-length of mRNA transcripts and their frequency of occurrence was determined. The positions and frequencies of cDNA variants derived from 5' RACE analysis are summarized in Fig. 1E. The major transcription start site of podoplanin mRNA was allocated to the position designated +1 (Fig. 1E and 2A).

### Isolation and structural analysis of the human *PDPN *promoter

The human *PDPN *gene 5' flanking region was isolated by PCR amplifications from a genomic BAC clone and subsequent staggered cloning. When comparing the isolated promoter sequence with the human chromosome 1 sequence, sequence identity was confirmed despite a single base difference at bp -415 (C in BAC clone versus A in the database). DNA sequence analysis using the Transfac database showed that the 5' flanking region lacked a consensus TATA box, whereas a GATA box (GATAAA) was localized 31 nucleotides upstream of the main mRNA transcription initiation site (Fig. 2A). The *PDPN *gene 5'-flanking region was characterized by a high GC content and revealed the existence of several potential Sp1 transcription factor sites. Moreover, putative binding sites for the basic transcription factors AP-2, AP-4, C/EBP, and NF-1, but not for osteoblast-specific factors were detected along the isolated *PDPN *promoter sequence (Fig. 2A).

### The *PDPN *promoter is transcriptionally regulated in MG63 and Saos-2 cells

Genomic DNA sequencing of the promoter sequence in Saos-2 cells confirmed that lack of *PDPN *transcription was not due to deletion mutations (data not shown). Thus, MG63 and Saos-2 cells proved to be a suitable cell system for investigation of transcriptional podoplanin regulation. To assess which portions of the promoter were involved in regulation of podoplanin expression, reporter plasmids harboring progressive deletions from the 5' end of the bp -1885/+171 *PDPN *flanking region were set according to putative binding sites of transcription factors (Fig. 2A). The sixteen promoter fragments were inserted upstream of a luciferase reporter gene (Fig. 2B) and were evaluated in transient expression analyses. Luciferase transcription was consistently 30 times more active in MG63 than in Saos-2 cells (Fig. 2C). In MG63 cells, bp -1104/+171 construct provided highest promoter activity (761 Luc/Ren × 100), whereas Saos-2 cells exhibited highest normalized luciferase activity with bp -498/+171 construct (27 Luc/Ren × 100). Transcriptional activity diminished progressively with further deletion of the 5' region, showing three activity minima with the constructs bp -666/+171, -261/+171 and -76/+171 in MG63 cells, and with constructs bp -186/+171 and -76/+171 in Saos-2 cells, which indicated the loss of positive regulatory elements (Fig. 2C). The low promoter activity of the smallest bp -26/+171 construct in both cell lines suggested that the 171 bp spanning 5'-UTR of podoplanin mRNA was not involved in upregulation of promoter activity. These data showed that the 1028 bp 5' region contained motifs providing basic *PDPN *promoter activity, whereas rather silencing motifs bound to the more upstream bp -1885/-1104 region.

### Nuclear proteins of MG63 and Saos-2 cells bind to the *PDPN *promoter

**Figure 3:**
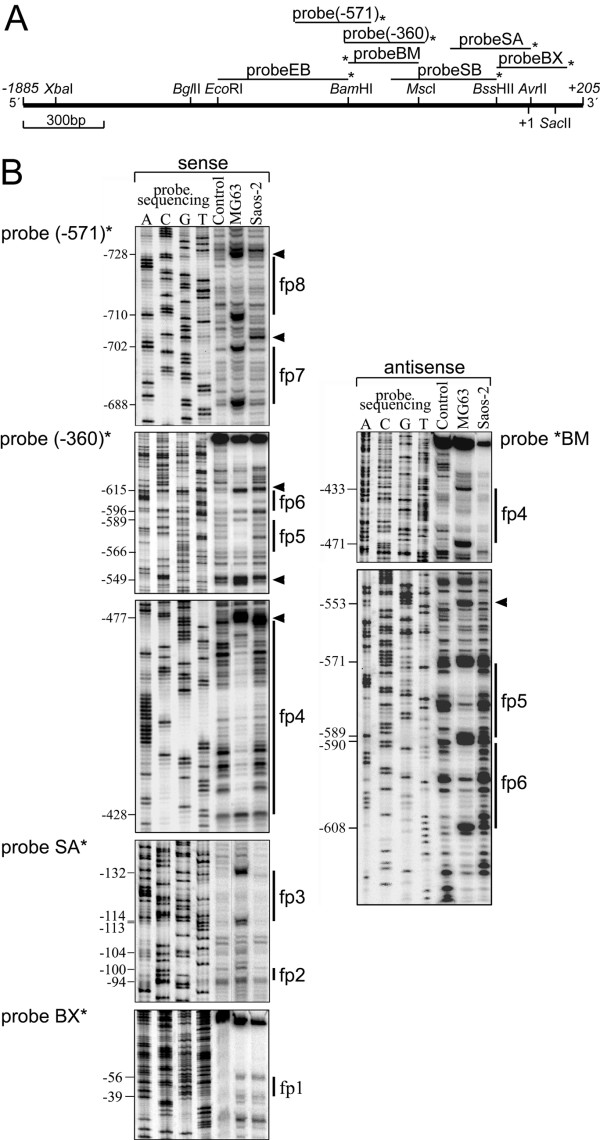
***In vitro *DNaseI footprint analysis of protein-binding to the *PDPN *promoter**. A) DNA probes used in DNaseI footprinting depicted above a restriction map of the -1885/+205 bp *PDPN *5'-genomic region. These were: probeEB (bp -1169 to -646), probe(-571) (bp -857 to -571), probe(-360) (bp -666 to -360), probeBM (bp -649 to -385), probeSB (bp -498 to -85), probeSA (bp -261 to +31), and probeBX (bp -89 to +171). *Asterisks *indicate the positions of radioactive labeling as described in the experimental procedures section. Six of the probes analyze the coding DNA strand, whereas probe BM tracks the noncoding strand. B) DNaseI footprinting of probes using MG63 and Saos-2 nuclear cell extracts. End-labeled restriction fragments were incubated alone (Control) or with MG63 or Saos-2 cell nuclear extracts prior to digestion with DNase I. Footprinting was carried out in the sense and antisense direction. A sequencing reaction of the same region was run in parallel for exact size assignment of the protected regions. Protected sequences are indicated with *single lines*. *Arrowheads *denote sites hypersensitive to DNaseI.

To more precisely define *cis*-acting DNA sequences responsible for cell-type specific *PDPN *transcription, seven overlapping DNA probes encompassing bp -1169 to +171 of the *PDPN *promoter (Fig. 3A) were used for *in vitro *DNaseI footprinting assays. With MG63 nuclear extracts, eight protected regions, fp8 – fp1, were identified with five probes (Fig. 3B), while probes EB and SB exhibited no differential DNaseI digestion pattern (data not shown). Complexes fp6, fp5 and fp4 were detected on both the coding and the noncoding DNA strand (Fig. 3B), and footprinting region fp1 became also visible with Saos-2 nuclear extracts (Fig. 3B). Moreover, digestional modified regions flanking the protected regions emerged along the probes (Fig. 3, arrowheads), additionally indicating the presence of DNA-binding proteins.

**Figure 4:**
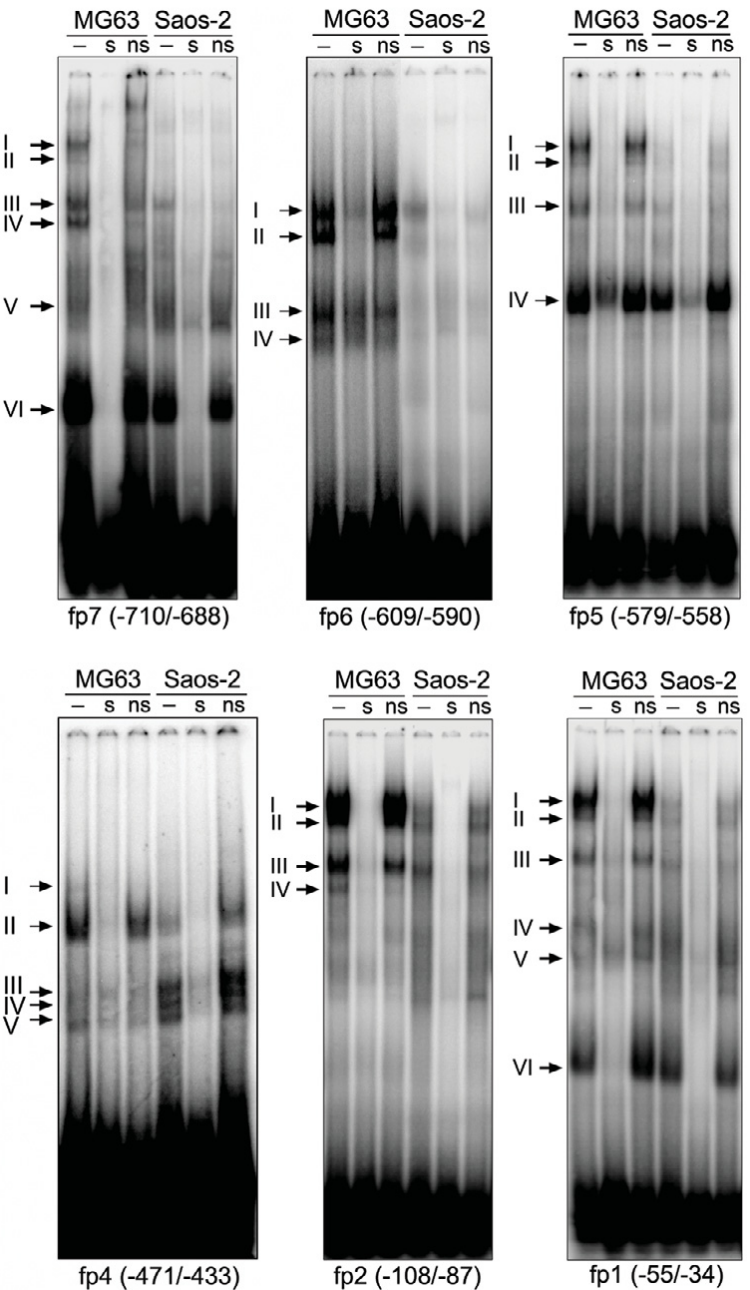
**EMSA analysis of footprint regions**. Sequence-specific proteins bound to oligos fp1, fp2, fp5 and fp7 of the human *PDPN *promoter. Nuclear extracts from MG63 and Saos-2 cells were incubated with ^32^P end-labeled double-stranded oligonucleotides, in the absence (-) or presence of a 100-fold molar excess of unlabeled specific (s) and unspecific (ns) oligonucleotide, as described in the "Methods" section. As unspecific probes, Oct-1 (fp1, fp2, fp5, fp7) or AP-4 (fp4 and fp6) consensus oligos were applied. *Arrows *denote the bands of altered mobility resulting from the interactions of nuclear proteins with the oligonucleotides.

To determine whether the detected elements indeed bound nuclear proteins, gel shift assays were performed using eight double stranded DNA oligonucleotides spanning the DNase I footprint regions (Table 1). Probes alone produced no shifted bands (data not shown). Probes fp7, fp5, fp2 and fp1 produced a similar pattern of low-mobility DNA-protein binding (complexes I – III) when adding nuclear extract of MG63 cells (Fig. 4, lanes -). The same bands became faintly visible with Saos-2 extracts except for probe fp7, which did not show complexes I and II. Consistent with the footprints, oligos fp6 and fp4 bound nuclear proteins strongly from MG63, but weak from Saos-2 cells (Fig. 4, lanes -). Although covering a broad footprint region, probe fp4 only yielded a binding pattern of several rapidly migrating proteins, and fp8 and fp3 did not reveal significant DNA-protein complexes (data not shown).

Specificity of the gel shifted bands was demonstrated by competition assays in which nuclear extracts were preincubated with a 100-molar excess of unlabeled specific (Fig. 4, lanes s) or unrelated probe (Fig. 4, lanes ns) as competitor. As all bands of the gel shifts were eliminated or attenuated by excess of unlabeled probe, these moieties may bind the respective oligos in a manner requiring concomitant association. A computer analysis revealed consensus motifs for transcription factors NF-1 (fp7, fp6), C/EBP-α (fp5, fp4), HNF-3 (fp4), Oct-1 (fp4) and AP-4 (fp1) in the applied probes. However, a panel of antibodies against these factors did not interfere with the observed low molecular weight DNA-protein complexes in supershift assays (data not shown). Nonetheless, these data suggested the presence of several sequence-specific proteins bound to the proximal *PDPN *promoter in MG63 cells.

### Sp proteins bind to fp7, fp5, fp2 and fp1in the *PDPN *promoter

**Figure 5:**
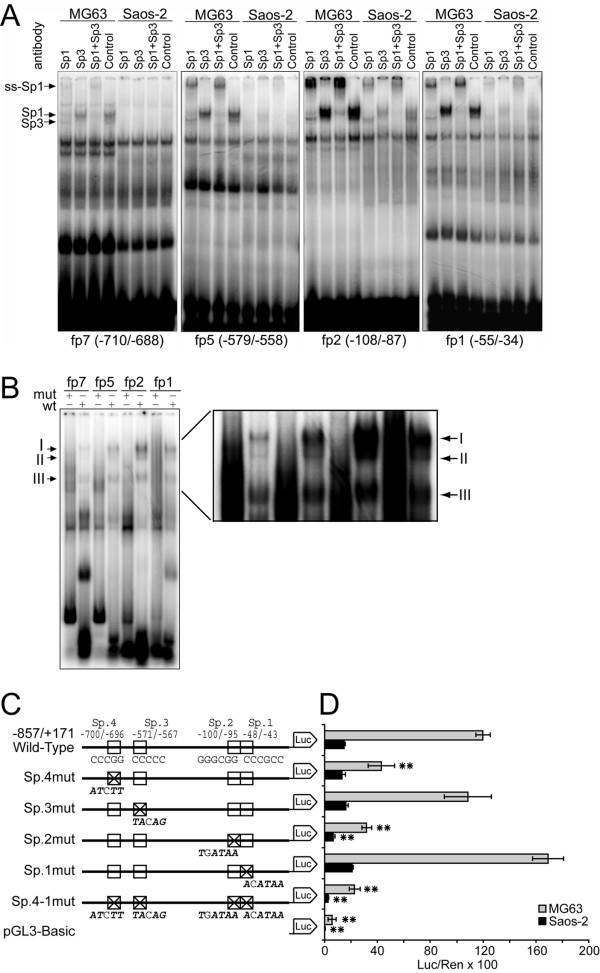
**Sp1 and Sp3 interact with the proximal *PDPN *promoter *in vitro *and *in vivo***. A) Supershifts were performed by incubating nuclear proteins either with anti-Sp1 antibody or with anti-Sp3 antibody, both antibodies together, or non immune IgG before addition of probe. Anti-Sp1 antibody supershifted complex I (ss-Sp1), while anti-Sp3 antibody specifically blocked the formation of protein-DNA complex II. B) Mutation of Sp binding sites (mut) abolished the binding complexes I and II versus the wild-type probe (wt) with nuclear extracts from MG63 cells. The mutated oligonucleotides used in this study are listed in Table 1. C) Deletion mutants of the four Sp-elements in the *PDPN *promoter. The sequences above the Wild-Type construct exhibit the core sequences of the Sp binding sites. Above each mutation construct, the respective mutated nucleotides are drawn in bold italics. In construct Sp.4-1mut, all four Sp elements are mutated. D) The wild type reporter construct and five mutation constructs were transiently transfected into MG63 and Saos-2 cells. Firefly luciferase values were divided by those of the internal control *Renilla *luciferase to represent the absolute promoter activity. Stars indicate the statistical difference of the promoter activities between the unmutated and mutated transfection experiments. *, p < 0.05; **, p < 0.01.

Probes fp7, fp5, fp2 and fp1 contained GC-rich regions exhibiting high homology to the consensus Sp1-binding motif. To test the binding of Sp-proteins to these elements, supershift analyses with anti-Sp1 and anti-Sp3 antibodies were performed. When anti-Sp1 antibody was added, complex I disappeared in favor of a supershifted band, which showed the predominant contribution of Sp1 (Fig. 5A, lanes Sp1). Addition of anti-Sp3 antibody blocked protein-DNA binding, which resulted in loss of complex II, whereas no supershifted complex appeared (Fig. 5A, lanes Sp3). When used together, Sp1 and Sp3 antibodies supershifted or inhibited both complexes (Fig. 5A, lanes Sp1+Sp3). To further test whether the core Sp1 sequence was required for binding of nuclear proteins, gel shift assays with the same oligonucleotide probes, but mutated at residues known to be critical for Sp-binding (Table 1), were performed with nuclear extracts of MG63 cells. The mutant oligos failed to form the observed complexes, indicating that the chosen mutations were sufficient to eliminate the binding capacities of these sites (Fig. 5B). These studies demonstrated that four elements within the *PDPN *promoter, herewith designated Sp.4 – Sp.1, bound Sp1/Sp3 proteins from MG63 and also weakly from Saos-2 cells. The results furthermore indicated that Sp1 and Sp3 were not bound coordinately to the DNA stretches, since the anti-Sp1 antibody did not shift Sp3-DNA complexes and *vice versa*. This suggested that Sp1 and Sp3 occupied the same or overlapping sites of the *PDPN *promoter.

### GC boxes Sp.4 and Sp.2 are essential for PDPN promoter activity

To assess the functional role of Sp binding elements Sp.4 – Sp.1, PCR-directed mutagenesis was performed at the same residues that had blocked Sp1/Sp3 binding in gel shift assays and were tested for their reporter activity. The four Sp binding sites were altered individually and in combination (Fig. 5C), and mutations were introduced into the promoter luciferase variant -857/+171 that supported basal *PDPN *transcription. The transactivation pattern was similar for MG63 and Saos-2 cells, indicating that Sp1/Sp3 promotion took place through the same elements in both cell lines (Fig. 5D). The utmost relevance was seen for Sp-binding site Sp.2 at bp -100/-95, showing an activity reduction by 73% in MG63 and by 52% in Saos-2 cells upon mutation. Deletion of Sp-binding site Sp.4 at bp -700/-696 decreased luciferase activity by 57% in MG63 and by 16% in Saos-2 cells, whereas mutation of Sp-binding site Sp.3 (bp -570/-564) did not alter luciferase activity substantially. Mutation of site Sp.1 (bp -48/-43) lead to a slight activating trend, while combined mutation of all four sites reduced activity to more than 80% in both cell lines. Taken together, these data suggested that Sp-binding sites Sp.4 and Sp.2 alone, but especially in concert, were central regulatory elements necessary for constitutive *PDPN *promoter activity in both MG63 and Saos-2 cells, whereas Sp.3 and Sp.1 provided no single activating importance.

### Stimulatory Sp1 and repressive Sp3 coordinatively activate the *PDPN *promoter

**Figure 6:**
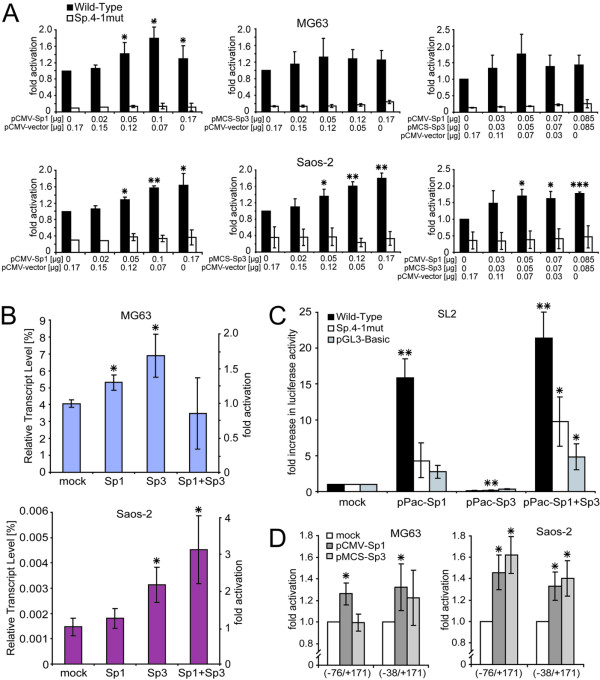
**Sp1 and Sp3 are critically involved in *PDPN *promoter activation**. A) Cotransfection of the core promoter construct (-857/+171) without (Wild-Type) and with Sp-binding site mutations (Mutant) using increasing amounts of pCMV-Sp1 and pMCS-Sp3 or a combination of both expression plasmids along with pRL-TK into MG63 and Saos-2 cells. The total amount of expression plasmids was filled to 0.17 μg with empty vector. Luciferase values were normalized to the internal control *Renilla *luciferase and fold-induction versus mock-transfected cells was evaluated. B) Real-time PCR analysis of podoplanin transcription in MG63 and Saos-2 cells upon transfection with Sp1 and Sp3 expression vectors. Transcript levels are relative to GAPDH mRNA content. The average ± S.D. from two independent experiments performed in triplicates is shown. C) *Drosophila *SL2 cells were transiently transfected with Wild-Type promoter, Sp.4-1mut construct and pGL3-Basic. Cells were cotransfected with *Drosophila *expression vectors pPac-empty, pPac-Sp1, pPac-Sp3, or both. Fold promoter activation versus pPac-empty transfections was evaluated. The average ± S.D. from three independent experiments performed in triplicates is shown. D) The role of Sp.1 for *PDPN *promoter activity. Cotransfection of the shortened promoter constructs (-76/+171) and (-38/+171) with pCMV-Sp1 or pMCS-Sp3 along with pRL-TK into MG63 and Saos-2 cells. The statistical difference of the promoter activity in all panels is shown against mock-transfected cells. *, p < 0.05, **, p < 0.01, ***, p < 0.001.

To directly confirm the effect of Sp1 and Sp3 on *PDPN *promoter activation, bp -857/+171 wild-type promoter luciferase construct was cotransfected with increasing amounts of CMV-promoter driven Sp1 and Sp3 expression plasmids alone and in combination into MG63 and Saos-2 cells (Fig. 6A). In both cell types, Sp1 overexpression resulted in a weak but dose dependent stimulation of luciferase activity with 1.8-fold induction in MG63 and 1.6-fold induction in Saos-2 cells relative to Sp1 mock-transfected cells. Similarly, Sp3 overexpression lead to a 1.4-fold and 1.8-fold induction in MG63 and Saos-2 cells, respectively. When both factors were coexpressed, no further activation above 1.8-fold promoter activity was seen. In contrast, luciferase activity of the combined mutational variant Sp.4-1mut was not altered in these experiments. These results suggested that Sp1 and Sp3 were able to stimulate *PDPN *transcription through Sp1/Sp3 binding sites in the proximal promoter region independently.

To confirm the effect of Sp1 and Sp3 on the activity of endogenous *PDPN *gene expression in MG63 and Saos-2 cells, real-time PCR was performed after transfection with Sp1- and Sp3-expression plasmids. The results corresponded to the observed potency of the respective factors to stimulate promoter activity. Podoplanin mRNA contents were clearly increased by overexpressed Sp1 and Sp3 (Fig. 6B). Overexpression of both, Sp1 and Sp3, did not further change mRNA levels in MG63 cells but strongly increased them in Saos-2 cells. These results showed that Sp1 and Sp3 indeed were able to upregulate podoplanin expression.

The roles of Sp1/Sp3 function on the *PDPN *promoter were more clearly established in *Drosophila *SL2 cells, which lack endogenous Sp-proteins. *Drosophila*-specific pPac-Sp1 and pPac-Sp3 expression plasmids were cotransfected with the wild-type and Sp-site mutant promoter variant Sp.4-1mut (Fig. 6C) and fold promoter activation versus pPac-empty transfections was evaluated. Cotransfection of Sp1 lead to a 16-fold luciferase activity increase of the wild-type promoter. In contrast to the results seen with MG63 and Saos-2 cells, overexpression of Sp3 reduced promoter activity 8-fold, which discovered a strong repressive action of solely present Sp3. Nevertheless, coexpression of both, Sp1 and Sp3, further increased promoter activity up to 21-fold. Since Sp1 activity was not decreased in the presence of pPac-Sp3, Sp3 seemingly did not compete with Sp1 for binding to the same site. The same effects were visible on a reduced level with mutant variant Sp.4-1mut and pGL3-Basic, indicating background activity of these constructs. Basically, these results suggested opposed action of single Sp1 or Sp3 on the *PDPN *promoter, while presence of both factors caused a concerted activation.

Mutation of site Sp.1 at bp -48/-43 (Sp.1mut) showed a slight increase in promoter activity (Fig. 5D), which corresponded to lowered activity of bp -76/+171 versus bp -38/+171 deletion construct (Fig. 2C). In order to explore a potential repressive role of Sp3 on this site, reporter constructs bp -76/+171 and -38/+171 were cotransfected with Sp1 and Sp3 expression plasmids and the multiple of promoter activation was determined (Fig. 6D). Consistently with luciferase reporter assays (Fig. 2C), construct -38/+171 conferred a roughly 2-fold higher promoter activity than -76/+171 in both cell lines (data not shown). Sp1 activated both constructs up to 1.4-fold in both cell lines. Consistent with the overexpression studies shown in Fig. 6A, Sp3 overexpression did not exert repressive effects on the bp -76/+171 construct. In MG63 cells, activity of the bp -76/+171 construct was not altered by additional Sp3-overexpression, while it even increased in Saos-2 cells. Therefore, we were not able to demonstrate a repressive Sp3 action on site Sp.1. We rather suggest a suppressive interaction of Sp-proteins bound to Sp.1 in combination with another yet unknown factor located more downstream from bp -38, which is abolished through shortening of the promoter or by deletion mutation of site Sp.1. A more detailed analysis of this site will be of future interest.

Conclusively, these results clearly demonstrated the pivotal importance of Sp1 and the auxiliary importance of Sp3 for basal transactivation of the *PDPN *promoter.

### Saos-2 cells exhibit reduced nuclear Sp protein levels and low Sp protein amounts bound to the *PDPN *promoter *in vivo*

**Figure 7:**
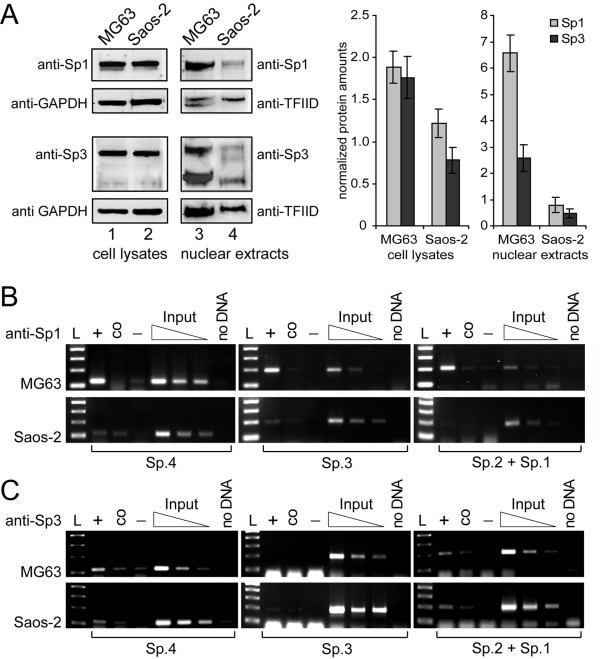
**Sp1/3 cellular versus nuclear levels and *in vivo *occupation of the *PDPN *promoter**. A) Immunoblot analysis and densitometric quantification of Sp1 and Sp3 protein contents in MG63 and Saos-2 cell lysates and nuclear extracts. Total cellular protein and nuclear extracts from MG63 and Saos-2 cells were resolved on 10% SDS-PAGE and blotted onto nitrocellulose. Levels of endogenous Sp1 and Sp3 proteins were determined by probing the blots with anti-Sp1 and anti-Sp3 antibodies, respectively. Sp1 shows a 105 kDa band, whereas Sp3 shows two bands at 120 and 75 kDa. Blots were reprobed with anti-GAPDH and anti-TFIID antibodies as loading control. After densitometric evaluation, Sp1/Sp3 levels were normalized to respective GAPDH and TFIID levels from the same blot. Data are given as the mean ± S.D. of samples of the experiment repeated three times. B) and C) Association of Sp1 and Sp3 to the *PDPN *promoter *in vivo*. Formaldehyde crosslinked chromatin was precipitated with an antibody specific for Sp1 (panel B) and Sp3 (panel C). PCRs were performed with primer pairs flanking the four Sp binding sites. L, 100 bp DNA ladder; +, immunoprecipitation with anti-Sp1 or anti-Sp3 antibody; co, precipitation control with unspecific rabbit IgG; -, negative control without antibody; Input, dilution series of control input DNA; no DNA, PCR negative control.

To further clarify the transcriptional difference between MG63 and Saos-2 cells, we focussed on the observation that nuclear extracts of Saos-2 cells produced weak Sp1/Sp3 EMSA signals compared to those derived with MG63 extracts (Fig. 5B). A comparative Western blot analysis showed little difference in overall Sp1/Sp3 protein contents between MG63 and Saos-2 cells (Fig. 7A, lanes 1 and 2, and densitometric analysis), whereas nuclear Sp1 and Sp3 amounts were lowered about 82 and 88%, respectively, in Saos-2 versus MG63 cells (Fig. 7A, lane 3 and 4, and densitometric analyis).

In order to assess whether Sp proteins bound to the identified DNA stretches *in vivo*, chromatin immunoprecipitations were performed using antibodies against Sp1 and Sp3 and crosslinked chromatin from MG63 and Saos-2 cells (Fig. 7B). After immunoprecipitation with anti-Sp1 antibody, extensive PCR amplifications from the Sp-binding sites relative to control input DNA were seen with MG63 derived chromatin (Fig. 7B, upper panels). In contrast, Saos-2 cells exhibited weak amplification levels versus input DNA (Fig. 7B, lower panels), which indicated low Sp1 occupation of these positions. PCRs from anti-Sp3 antibody precipitated chromatin resulted in overall weaker amplification results (Fig. 7C), which corresponded to the low DNA-bound Sp3 amounts seen in EMSAs (Fig. 5A). Site Sp.4 was stronger occupied by Sp3 in MG63 than in Saos-2 cells, while in both cell types site Sp.3 was scarcely occupied by Sp3. Sites Sp.2 and Sp.1 were acquainted with Sp3 to an equal, yet moderate extent in both cell lines. These data raised the possibility that drastically reduced nuclear Sp protein concentrations were responsible for weak *PDPN *promoter activity in Saos-2 compared to MG63 cells.

### The *PDPN *promoter is strongly methylated in MG63 but not in Saos-2 cells

**Figure 8:**
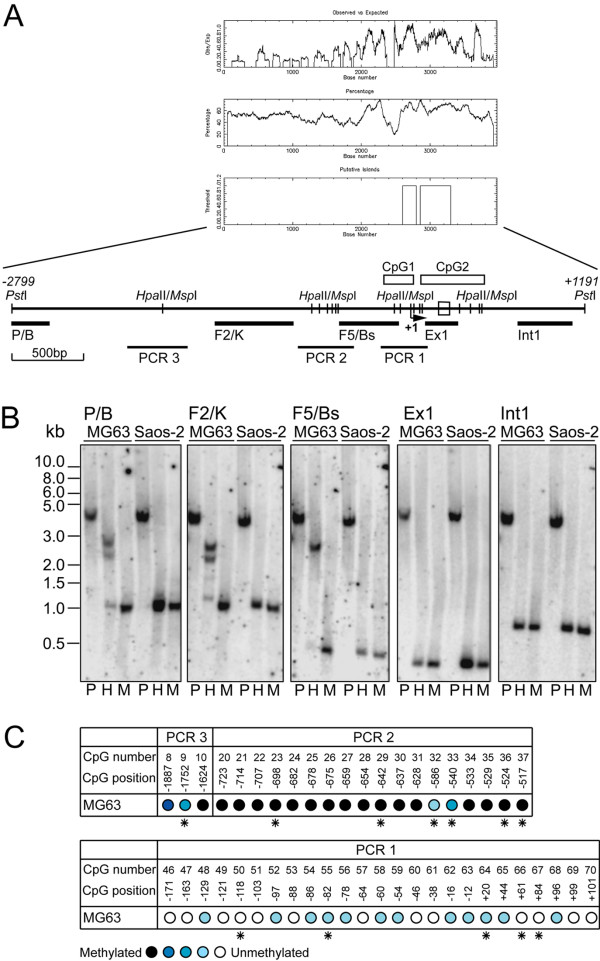
**The distal *PDPN *promoter is fully methylated in MG63 but non-methylated in Saos-2 cells**. A) Schematic representation of *Hpa*II/*Msp*I binding sites and CpG islands within a *Pst*I genomic DNA fragment spanning from bp -2799 to +1191 of the *PDPN *promoter with respect to the transcription initiation start site (arrow at +1). The graphs depict the CpG blot created by the EMBOSS program. Exon 1 is drawn as an open box in the sequence. *Hpa*II/*Msp*I recognition sites are represented by vertical lines along the sequence, and CpG islands are indicated as empty boxes above. Probes used for Southern blot analysis and stretches that were amplified after bisulfite genomic DNA sequencing are indicated as black bars and lines, respectively, below the sequence. B) Genomic DNA from MG63 and Saos-2 cells was double-digested with *Pst*I, and with *Pst*I in combination with the methylation-sensitive restriction enzyme *Hpa*II or the methylation-insensitive restriction enzyme *Msp*I. DNA then was fractionated by agarose gel electrophoresis followed by Southern blotting using the five DNA probes depicted in Fig. 8A. C) CpG methylation pattern of the *PDPN *promoter and of exonic and intronic CpG islands in MG63 cells as determined by sodium bisulfite genomic DNA sequencing. The CpG number indicates the order of the CpG site inside the *Pst*I genomic fragment, and the CpG position denotes its position in relation to the transcription start site. The color-tone of the circle reflects the degree of methylation: black: 100%, dark blue: 99-67% blue: 66-34%, light blue: 33-1%, white: 0% methylation. *Asterisks *indicate CpG motifs that are part of a *Hpa*II/*Msp*I restriction digest site.

To further unravel the transcriptional difference between podoplanin expression in MG63 and Saos-2 cells, epigenetic effects were considered. The *PDPN *promoter contained a high CG content which provided putative methylation sites. Correspondingly, two CpG islands were detected [28], one encompassing bp -183/+16 that spanned the core promoter, and the other stretching from bp +76 to +517, including exon 1 (bp +203 to +267) plus 249 bp of intron 1 (Fig. 8A). A *Pst*I genomic DNA-fragment that covered bp -2799 to +1191 of the *PDPN *promoter and gene was used to analyze a potential methylation-sensitive restriction pattern. The fragment contained 121 palindromic CpG dinucleotides, sixteen of which represented isoschizomeric *Hpa*II/*Msp*I restriction sites accumulating in three clusters. Five Southern blotting probes were situated beside and between these clusters (Fig. 8A). Digestion of genomic DNA with *Pst*I alone produced the anticipated 3990 bp fragment (Fig. 8B, lanes P), and double-digestion with *Pst*I plus methylation-insensitive enzyme *Msp*I yielded the smallest possible fragments (1047, 1044, 399, 257 and 700 bp, respectively) (Fig. 8B, lanes M). When using *Pst*I together with methylation-sensitive *Hpa*II, complete digested bands were observed with Saos-2 derived genomic DNA, while in MG63 cells the distal probes P/B and F2/K mainly detected undigested 2.2 and 2.7 kb bands besides small portions of complete digested fragments (Fig. 8B, lanes H). Moreover, with probe F5/Bs only the digestion resistant 2.7 kb fragment was visible. These results indicated strong methylation of region bp -2799/-464 in MG63 cells, while it was totally unmethylated in Saos-2 cells.

To clarify the *PDPN *promoter methylation status in MG63 cells in detail, genomic DNA was analyzed by bisulfite sequencing. Genomic DNA was treated with sodium bisulfite under conditions where cytosines are converted to uracils, while methylated cytosines remain unmodified [29]. Three PCR reactions were set to inspect the methylation level of the *Hpa*II/*Msp*I restriction site clusters (Fig. 8C). When PCR1 (bp -198 to +131) was considered, the methylation level was found to be less than 5%. With PCR2 (bp -759 to -485), except CpG sites 32 and 33 that were methylated to 25% and 50%, respectively, all CpG sites were fully methylated. PCR3 (bp -2005 to -1572) comprised three CpG sites, which were highly (site 8 to 86%, site 9 to 57%) or fully (site 10) methylated. The observed methylation pattern corresponded to the fragmentation results seen in Southern blotting and identified a highly methylated DNA status of the *PDPN *promoter upstream of bp -485 in MG63 cells.

### Methylation of GC boxes Sp.4 and Sp.2 does not affect Sp-protein binding

**Figure 9:**
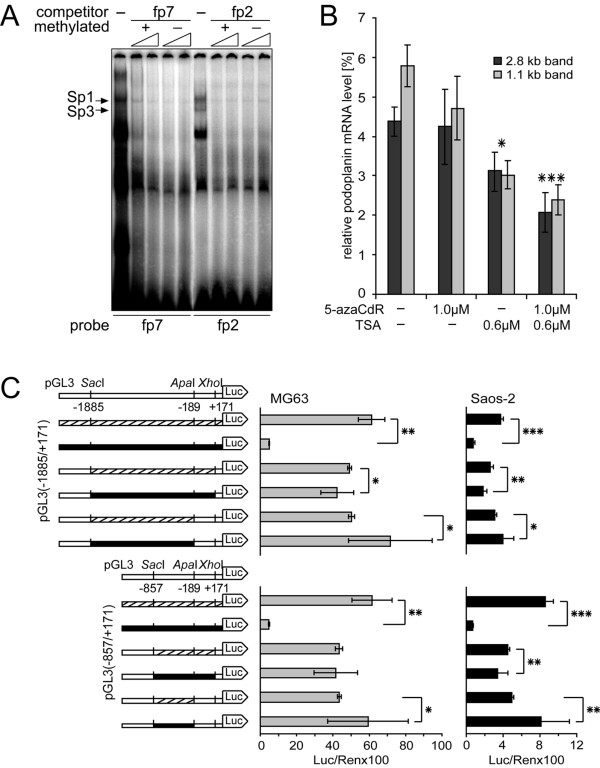
**The role of *PDPN *promoter methylation in MG63 cells**. A) EMSA competition experiments were performed using cold methylated and unmethylated fp7 (site Sp.4) and fp2 (site Sp.2) probe as competitors. Competitors were used at 50-fold excess at the small end of the triangle and at 100-fold excess at its large end. B) Densitometric quantification of both podoplanin mRNA transcripts isolated from MG63 cells which had been treated with 5-azaCdR and TSA alone or in combination. The abundance of podoplanin transcripts was normalized to GAPDH transcripts in the same samples. The data are given as the mean ± S.D. of triplicate samples of the experiment repeated twice. The statistical analysis denotes the significant difference between non-treated MG63 cells and cells treated with 5-azaCdR or/and TSA. *, p < 0.05, ***, p < 0.001. C) Whole pGL3-constructs or selected inserts thereof were methylated or mock-methylated *in vitro *and transfected into MG63 and Saos-2 cells. Results are given as luciferase activity normalized to cotransfected pRL-TK activity. The experiment was repeated three times independently in triplicates. The statistical analysis denotes the significant difference between mock methylated (hatched) and respective methylated (black) constructs. *, p < 0.05, **, p < 0.01, ***, p < 0.001.

In MG63 cells, the heavy methylation pattern stretched accross functional Sp1/Sp3 binding site Sp.4 (CpG number 23, Fig. 8C), whereas binding site Sp.2 was situated in the virtually unmethylated proximal promoter region (CpG number 52, Fig. 8C). To test whether this situation gave rise to modulatory binding of Sp-proteins, we compared the capabilities of respective oligos fp7 (site Sp.4) and fp2 (site Sp.2) to inhibit the formation of Sp1-/Sp3-DNA complexes with nuclear extracts of MG63 cells in methylated as well as unmethylated form. As shown in Fig. 9A, protein-DNA complexes were competed by increasing concentrations of both, methylated and unmethylated forms of probes fp7 and fp2 to an equal extent. These data suggested that Sp binding sites Sp.4 and Sp.2 were able to interact with Sp1/Sp3 even when methylated.

### Distal promoter methylation enhances *PDPN *promoter activity

To further test the role of DNA methylation and histone acetylation for control of podoplanin expression *in vivo*, MG63 and Saos-2 cells were treated with methyltransferase inhibitor 5-aza-2'-deoxycytidine (5-azaCdR) and histone deacetylase inhibitor trichostatin A (TSA). Cell viability was tested by increasing concentrations of the agents over a cultivation period of 3 days to exclude mRNA downregulation effects upon apoptosis. Then, cells were treated either with 5-azaCdR and TSA alone or with 5-azaCdR followed by TSA, and podoplanin mRNA amounts were analyzed by quantitative Northern blot analysis. In Saos-2 cells, the drugs were not able to initiate *de novo *synthesis of podoplanin mRNA (data not shown), which demonstrated that lack of *PDPN *transcription was not under epigenetic control. In MG63 cells, variations of both podoplanin mRNA bands seen in Northern blot were evaluated (Fig. 9B). 5-azaCdR treatment did not change *PDPN *transcription levels when compared to untreated cells, while 0.6 μM TSA lead to a respective 28% and 48% reduction of *PDPN *transcription. Moreover, a combination of 1 μM 5-azaCdR and 0.6 μM TSA decreased *PDPN *transcription by 50% and 57%, respectively. These data indicated that in MG63 cells, *in vivo *modification of the promoter chromatin structure through combined demethylation and histone hyperacetylation rather repressed than enhanced *PDPN *transcription.

To investigate whether methylation of the distal *PDPN *promoter portion indeed assisted activation of gene transcription, further luciferase reporter gene assays were performed (Fig. 9C). Complete i*n vitro *methylation of the promoter plasmids pGL3(-1885/+171) and pGL3(-857/+171) lead to more than 90% loss of promoter activity in MG63 and Saos-2 cells, indicating that the vectors were susceptible to inhibition by methylation. To further assess promoter activity upon selective methylation of defined promoter regions, fragments bp -1885/+171 and -857/+171, or fragments bp -1885/-189 and -825/-189, which comprised the region that wasmethylated *in vivo *in MG63 cells, were generated by restriction digest with *Sac*I/*Xho*I or *Sac*I/*Apa*I, respectively (Fig. 9C). The four fragments were methylated by *Sss*I methylase or mock-methylated, religated into the unmethylated reporter plasmid backbone and transfected into MG63 and Saos-2 cells. *In vitro *methylation of all promoter CpGs until bp +171 revealed a weak trend towards transcriptional deactivation being more pronounced in Saos-2 than in MG63 cells (Fig. 9C). This result supported the observation that methylation of site Sp1.2 did not affect Sp1/Sp3 binding (Fig. 9A). Conversely, selective methylation of CpG sites upstream of bp -189 containing 35 or 24 CpG sites, respectively, led to considerable promoter activity increase versus mock methylated constructs, which added up to more than 40% and 30% in MG63 and to more than 25% and 60% in Saos-2 cells. These data indicated that a putative methylation-dependent enhancement mechanism, possibly efficiently altering chromatin structure, was able to act on *PDPN *transcription.

## Discussion

The present study provides a comprehensive analysis of the *PDPN *gene promoter region in human osteoblast-like cell lines. It was our aim to clarify which mechanisms account for cell type-specific expression of podoplanin in MG63 versus Saos-2 cells, as direct regulators of *PDPN *transcription in human systems have not been elucidated yet. We show that *PDPN *transcription was initiated from several start sites within 97 bp, the most 3' located transcript of which corresponded to the major initiation site in the murine and rat gene [24]. Generally, the *PDPN *promoter seems to belong to the group of regulative genomic entities which are characterized by the absence of a consensus TATA and CAAT box, by a high GC content and multiple potential binding sites for the transcription factor Sp1. Such genes are usually either ubiquitously expressed (e.g. housekeeping genes) or are growth related, as for instance dihydrofolate reductase [30]. Nonetheless, genes possessing these features have been identified to be expressed in tissue-specific manner, e.g. RAGE [31] or thioredoxin reductase 1 [32]. The chosen promoter fragments indeed conferred intense transcriptional activity in MG63 but not in Saos-2 cells, indicating the presence of differentially bound positive regulators.

Functional analyses of the proximal *PDPN *promoter revealed four potential Sp1/Sp3-binding sites and the key roles of two of these at bp -700/-696 (site Sp.4) and bp -100/-95 bp (site Sp.2) for constitutive activation in both, MG63 as well as Saos-2 cells. Sp1 has been the first mammalian transcription factor to be cloned [33] and invariably activates transcription [34], whereas Sp3, dependent on the position of the element within the intact promoter and the number of Sp binding sites [35], is able to either enhance or suppress transcription. Sp1 and Sp3 have been described to be involved in the regulation of osteoblast-specific genes like beta 5 integrin [36], osteocalcin [37], or alpha2 (XI) collagen (COL11A2) [38] yet. The role of the more distal site Sp.4, which was conserved in the rat promoter, has not been described elsewhere, whereas site Sp.3 was not conserved. Binding site Sp.2 was conserved between human, rodent and bos tauris *PDPN *sequence, and its activating role has previously been demonstrated in rat type I alveolar cells [24]. Also binding site Sp.1 was conserved between humans and rodents.

The effect of Sp protein transactivation on the *PDPN *promoter was seen pronounced in *Drosophila *SL2 cells. Interestingly, Sp1 and Sp3 seemed to regulate *PDPN *transcription in a contrarious, yet overall cooperative manner. The results demonstrated a strong positive effect of Sp1 on the *PDPN *promoter, whereas Sp3 acted as repressor. In MG63 and Saos-2 cells, a repressive effect of Sp3 was not observed, which, however, may be masked by concurring Sp1-binding through endogenously present Sp1. On the other hand, Sp protein overexpression effects were not very pronounced in these cells, presumably by the same reason of intrinsic Sp protein background. One could argue that Sp1 or Sp3 were not essentially important to control *PDPN *promoter activity, but when considering the generally low transfection rates of less than 10% in MG63 cells, an overall 1.8-fold promoter activation corresponded to a 12-fold induction of promoter activity if all cells were transfected. Sp3 stimulated the *PDPN *promoter in Saos-2 cells stronger than in MG63 cells, which can be explained through a more potent effect due to the lowered nuclear Sp3 amounts as seen from Western blotting. Additionally, this effect might have resulted from generally better transfection efficiencies in Saos-2 cells. Real-time PCR analyses then convincingly demonstrated the effect of Sp-proteins on endogenous *PDPN *gene expression in both cell lines. We therefore assume that a balance between cooperatively bound Sp1 and Sp3 tightly regulates activity of the *PDPN *promoter in these osteoblastic cell lines.

Sp1/Sp3 site EMSA analyses suggested that they were occupied by regulators mainly present in MG63, but not in Saos-2 cells. The faint Sp1/Sp3-DNA interactions of Saos-2 nuclear extracts may be due to several reasons: Firstly, nuclear Sp-proteins can be inactivated by dephosphorylation [39], or Sp-proteins may be present in the nuclei, but in very low concentrations. Indeed, Western blotting experiments pointed out that nuclear Sp1/Sp3 protein amounts were distinctively lower in Saos-2 than in MG63 cells, and chromatin immunoprecipitations confirmed the low *in vivo *occupancy of Sp binding sites in Saos-2 cells. Hence, these low Sp-protein concentrations might be insufficient to drive *PDPN *transcription and strikingly would explain the lack of podoplanin mRNA in Saos-2 cells. In MG63 cells, nuclear Sp1/Sp3 quantities were highly abundant, which could rely on increased nuclear Sp protein translocation and/or a decreased efflux of Sp proteins out of the nuclei [39]. Consistent with our results, hyperoxia treatment of murine lung epithelial cells caused an increased nuclear abundance of Sp1 that lead to enhanced expression of podoplanin/T1α [40].

Besides control through nuclear concentration thresholds, Sp-mediated transcriptional changes can be mediated by interactions with additional transcription factors, such as cAMP [41] or NFκB [42]. When reducing the *PDPN *promoter stretch from bp -1104/+171 to bp -854/+171, transcriptional activity decreased about more than 50%. In DNaseI footprintings, however, no protected region could be detected in this region. Proteins attaching to this region might need concurrent binding of Sp1/Sp3 or of other factors more downstream. Such interaction mode without direct DNA contact could also explain the divergent results of DNaseI footprints and EMSA analyses at regions fp8, fp6, fp4 and fp3. In contrast to the reproducible footprinting results, nuclear proteins binding directly to the same motifs could not be detected in EMSAs. This result may either be explained by the presence of low affinity factors that rapidly dissociate from the DNA during the assay, or the attachment of proteins to these sites may depend on adjacent bound Sp1/Sp3-proteins and consequently could not be grasped due to the restricted length of the applied DNA probes. Consecutively, in Saos-2 cells accessory assembly of the same activating factors would be impossible due to lack of Sp allocation.

Moreover, despite its conservation between humans and rodents, we could not clearly prove functionality of binding site Sp.1 through repressive Sp3. Surprisingly, the higher promoter activity of the bp -38/+171 versus bp -76/+171 variant, as well as of the mutational Sp.1 variant rather seemed to result from additional interactions to a factor located more downstream than through repressive Sp3. Possibly, this mechanism may be a regulative to suppress extensive *PDPN *transcription. For example, interaction of Sp1 with E2F has been shown to control growth related gene transcription [43]. Studies are ongoing that shall clarify the presence and identity of factors that are physically associated with Sp1/Sp3 bound to sites fp7, fp2 and fp1.

Sp7/Osterix respresents an interesting Sp protein family candidate relevant for activation of osteoblast-specific genes. It is essential for osteoblast differentiation and bone formation [44], and its expression has been detected in MG63 [45] as well as Saos-2 cells [38]. Its potential role for *PDPN *promoter activation may be of interest and shall be clarified in future studies. As in murine and human osteosarcoma cells, on the other hand, Sp7/Osterix expression has been detected to be reduced versus normal osteoblasts [46], we presume that Sp7/Osterix contents might also be lowered in MG63 and Saos-2 osteosarcoma cells and therefore would exert a minor effect on *PDPN *promoter activation.

To unravel the transcriptional difference between MG63 and Saos-2 cells more thoroughly, potential DNA methylation of the *PDPN *promoter was examined. We identified complete promoter demethylation in Saos-2 cells, although no *PDPN *transcription took place. By contrast, in MG63 cells the distal promoter part was strongly methylated, while the *PDPN *gene was efficiently transcribed. This DNA modification affected the functional Sp protein binding site Sp.4, but i*n vitro *competition data proved that Sp-protein binding to this site was possible even when it was methylated. Our data therefore strongly support studies demonstrating that CpG methylation has no effect on attachment of Sp1 [47], whereas others have found that it diminished Sp1/Sp3 binding [48].

**Figure 10:**
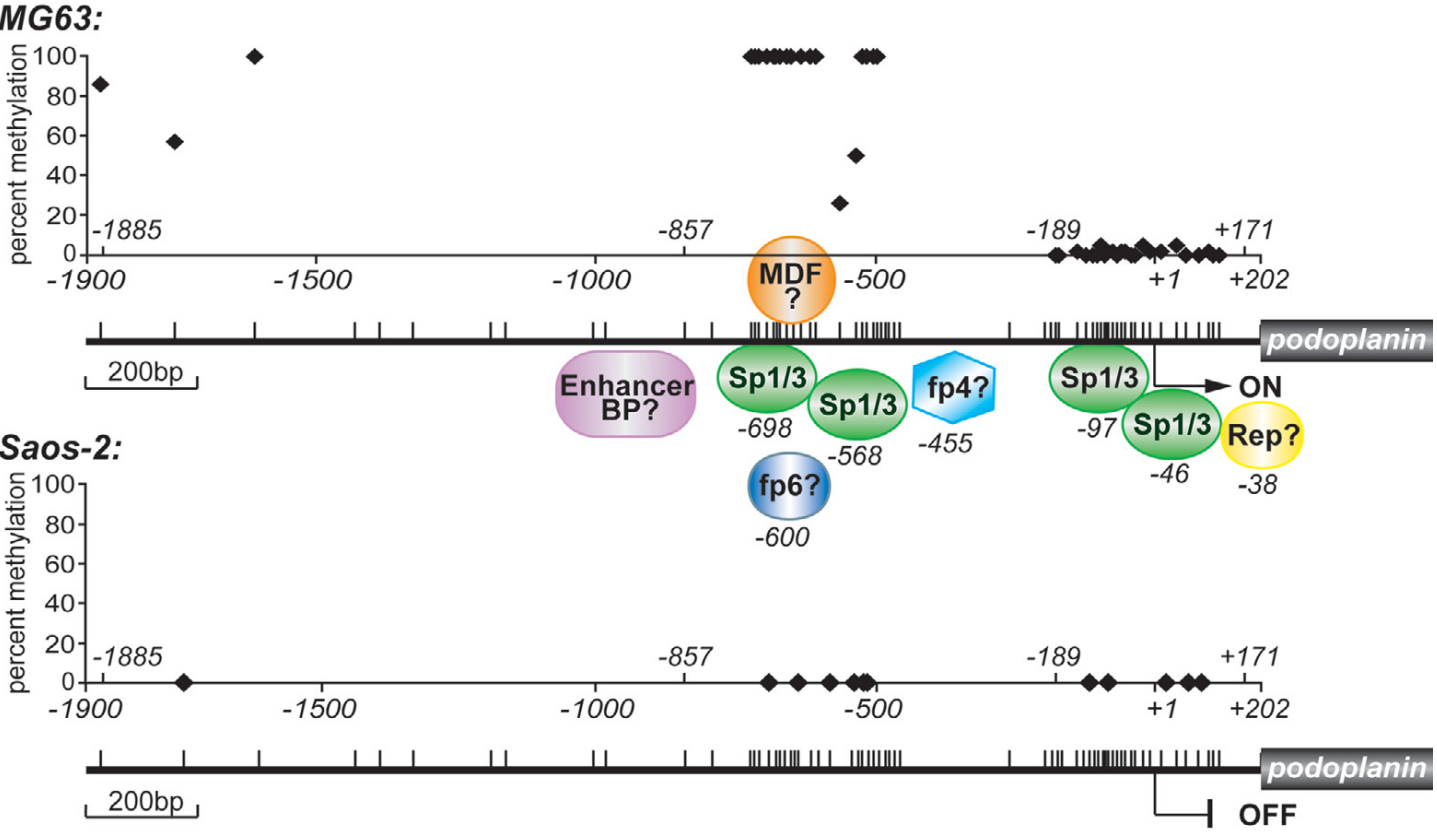
**Possible model of transcriptional control of podoplanin expression in MG63 versus Saos-2 cells**. The *PDPN *promoter is active, when, accompanied by distal hypermethylation, several factors accumulate near Sp1/Sp3 proteins bound at sites bp -700/-696 (Sp1.4) and bp -100/-95 (Sp1.2). A factor located downstream from bp -38 and associated with Sp1/Sp3 bound to site Sp.1 may exert a repressive effect. The *arrow *indicates the major transcription start site; the small vertical *bars *indicate the CpG sites; the *diamonds *indicate the CpG sites analyzed in this study; *fp*, footprint detected protein; *MDF*, methylation dependent factor.

Chromatin modification by CpG methylation and/or histone deacetylation has been linked inevitably with transcriptional repression [49]. However, our data provide evidence for a positive correlation between DNA methylation/histone modification and *PDPN *transcription in MG63 cells, as in our hands, induction of this classical "active chromatin state" rather conferred deactivation of gene activity. More importantly, site-specific DNA methylation by no means lead to down-modulation of promoter activity. The theoretical possibility of a repressor binding to the promoter and being competed by DNA methylation in MG63 cells has to be excluded, because demethylated *PDPN *promoter (i.e. the pGL3-promoter luciferase constructs) was highly active in MG63 cells. In agreement with that, no repressor-DNA interactions could be identified in Saos-2 cells. The mechanistic principle underlying the observed phenomenon could be diverse: When treating MG63 cells with 5-azaCdR in combination with TSA, consecutive conformational changes of chromatin may hinder Sp-protein binding and lead to downregulation of *PDPN *transcription. Secondly, it is tempting to hypothesize that methylation-dependent protein-DNA interactions being able to stimulate transcription may exist (methylation dependent factor *MDF*, Fig. 10). For example, the zinc finger protein Kaiso has been found to bind to both, methylated and sequence-specific recognition sites, fulfilling repressive [50] or activating functions [51]. Here, we identify a completely different methylation scenario than has been observed in brain and lung alveolar type I cells of the rat [25]. In these cells, DNA methylation was detected at key site Sp.2 under podoplanin/T1α non-expressing conditions, whereas its transcription could be initiated by addition of methyltransferase-inhibitor. These findings may indicate that the switch between transcriptional silencing and expression of podoplanin in different tissues/organs could depend on varying epigenetic phenomena, apparently driven by basal Sp1/Sp3 activation at conserved promoter sites. Studies using additional cell lines that constitutively or upon stimulation express podoplanin are ongoing to better understand the effect of methylation on this type of promoter. Preliminary data are pointing at again different methylation patterns in other cell lines. Moreover, altered histone modification patterns of the *PDPN *promoter chromatin have to be considered, as this process is intimately connected with DNA methylation [52]. Presumably, diverse regulatory pathways are mediating these epigenetic patterns in different cell types or even species.

To our knowledge, this investigation is only the second one reporting on a certain protein exhibiting increased transcription levels upon methylation [53]. Unlike podoplanin, this report describes increased transcription of an imprinted gene (*Igf2*) upon enhanced intragenic methylation, whereas in our study the 5' regulatory region was affected. Similar to our findings, some studies have shown a positive effect on gene expression when methylation downstream of the transcriptional start site had occurred [54], and hypermethylation of CpG islands has been detected in actively transcribed regions of cancer cells [55]. On the other hand, hypermethylated gene promoters, generally, have been associated with inappropriate gene silencing as an effect of selection during neoplasia [56], which follows a primary leakiness of the cellular repression machinery [57]. A further unexpected finding of our study is hypomethylation of an inactive promoter. In accordance with this, 1–2% of genes of murine fibroblast cultures observed in an array study were downregulated through genomic demethylation [58]. Moreover, hypomethylated inactive DNA has been detected throughout large areas of the inactive female X chromosome and in gene-poor genomic regions of tumour cells [56]. Recapitulatory, changes in DNA methylation patterns play a fundamental role in cancer and may regard to losses or increases of epigenetic patterns [59]. The epigenetic event observed in our study may have provided a selection advantage to MG63 osteosarcoma cells and further clonal selection, considering that podoplanin recently has been assessed to contribute to tumour progression by circumventing epithelial-mesenchymal transition [22].

## Conclusion

Conclusively, we studied the cell-type specific regulation of podoplanin expression in human MG63 versus Saos-2 osteoblast-like osteosarcoma cells. The results are summarized in the proposed activation model depicted in Figure 10.

Our findings indicate that Sp1/Sp3 members constitutively bind at least to three responsive elements of the *PDPN *promoter in MG63 cells *in vivo*, and that the activity of the promoter primarily depends on the integrity of two of these sites. Increased nuclear Sp1/Sp3 levels were shown to be responsible for activation of the promoter in MG63 versus Saos-2 cells. Additional transcription factor complexes located even more upstream seem to up-regulate *PDPN *gene expression strongly in MG63 versus Saos-2 cells. Analysis of the human *PDPN *promoter sequence undertaken here did not detect high score binding sites for osteoblast-specific transcription factors. However, the possibility that such factors participate in *PDPN *transcriptional regulation cannot be excluded, either by representing coactivators without direct DNA interaction, or by binding to far more upstream or downstream regulatory regions. A highly methylated chromatin conformation present in the distal promoter in MG63, but not in Saos-2 cells, leads to auxiliary enhancement of transcriptional activity of the *PDPN *gene. Our data are conflictive with previous findings supporting a negative correlation between methylation status and transcriptional activity of a promoter. Studies are under way to further dissect the mechanistic principle of regulation provided by this type of promoter in other podoplanin expressing cell lines.

## Methods

### Cell culture

Human osteoblast-like osteosarcoma cell lines MG63 and Saos-2 were obtained from American Type Culture Collection (ATCC CRL-1427 and HTB-85, respectively). Cells were maintained at 37°C with 5% CO_2 _and were grown as described in the ATCC protocol. *Drosophila *Schneider SL-2 embryo cells (ATCC CRL-1963), a kind gift from Dr. Hans Rotheneder, were maintained in Drosophila-SFM (Gibco) supplemented with 2 mM glutamine without antibiotics.

### Reverse transcription and Real-time PCR, Northern blotting and RACE

Total RNA was extracted from cells lines with TriReagent (Molecular Research Center, Inc.). For RT-PCR analysis of cell lines, total RNA of twelve different cell lines was transcribed into cDNA using RT-for-PCR-Kit (Clontech), and PCRs were carried out with Taq DNA Polymerase (Amersham Biosciences) and the RNA-specific primers 12B1-F1 and LF3-AT, which create a 322 bp PCR fragment. For real-time PCR, cDNA was synthesized with the Sprint PowerScript Kit (Clontech). PCR then was performed with the *PDPN*-specific primers Hs 01089982-g1 and the *GAPDH*-specific primers Hs 9999905-m1 (Applied Biosystems) in an ABI Prism 7000 Taqman. For analyzation of multiple potential start sites, total RNA was transcribed into cDNA by Superscript II RT (Invitrogen) using the *PDPN *gene specific 3' primer hPP (+207/+178) located at the 3'-end of the 5' untranslated region. RT-PCR was then performed with nine 5' primers (Table I) in combination with the 3' primer hPP (+114/+96). For Northern blotting, total RNA was fractionated on 1.2% formaldehyde-denaturing agarose gels, transferred onto a nylon membrane (Zeta-probe GT genomic membrane, BioRad) and fixed by UV cross-linking. Blots then were incubated with a probe covering bp +206 to +723 of podoplanin mRNA and with a GAPDH probe for normalization of RNA amount. Densitometric quantification was perfomed with the LumiAnalyst 3.0 program (Roche). 5'- and 3'-RACE were performed using respective RACE systems (Gibco) and total RNA derived from MG63 cells. PCR products derived from both procedures were cloned into vector pCR2.1 (Invitrogen) and the sequencing results of randomly picked clones were employed to construct a map of transcriptional variants.

### Isolation of the *PDPN *promoter

The genomic BAC clone 53F6 was derived from the Human RPCI-11 library (Research Genetics) [60]. By Southern blotting of several restriction digests, utilizing a unique *Sac*II site at bp +125 as a size reference point, the clone was assayed for containing a large portion of the 5'-upstream region of the human *PDPN *gene. 4.6 kb of the *PDPN *5'-flanking region were sequenced by primer walking applying the Sanger dideoxy chain termination method. According to these results, four subsequent PCR primer pairs were designed, which enabled the generation of four fragments each covering ~1.2 kb of the 5'-flanking region, and, by the insertion of appropriate restriction sites, stepwise cloning of the promoter. The PCR products were cloned separately into the T/A cloning site of pCR 2.1 (Invitrogen) and subjected to sequence analysis. Potential transcription factor binding sites were predicted using the Alibaba and Gene Viewer programs, which employ TRANSFAC 5.0 matrices [61] with a core similarity of 1.0 and matrix similarity 0.9. Potential CpG islands were identified by the EMBOSS CpGplot program package [62].

### Plasmid constructs

Two *PDPN *promoter constructs were derived from the original cloning procedure with 5' primers FII, FI and 3' primer RI (Table 1). Additional deletions were prepared using fourteen 5' primers (Table 1) in combination with 3' primer RI. PCR products were generated by standard PCR procedure and cloned into the T/A cloning site of the pCR 2.1 vector. 5'-flanking *Sac*I and *Xho*I sites, which had been synthesized at the upper and lower primers, were used for unidirectional cloning of fragments upstream from the luciferase gene into the pGL3-Basic vector (Promega). Mutations at Sp1/Sp3 consensus sites were introduced using mutation primers (Table 1) and the QuikChange II site-directed mutagenesis kit (Stratagene). A Sp1 expression plasmid was derived from cDNA clone Nr. 7030772 (ATCC, Molecular Genomics Resources), and a loss of function mutation in this clone was corrected by site directed mutagenesis as described above. After digestion with *Xma*I, the Sp1 coding region was cloned into the correct reading-frame of expression vector pIRES-neo (Stratagene), which contains a CMV promoter. pMCS-Sp3, pPac-Sp1, pPac-Sp3 and pPac-empty were a kind gift from Dr. Guntram Suske, Marburg, Germany [63]. All DNA constructs were purified using Endo-free Midiprep Kit (Promega), and correct identities were confirmed by sequencing.

### Transient transfections and luciferase reporter assay

MG63 and Saos-2 cells were transiently transfected in 24-wells using Effectene transfection reagent (Qiagen), as recommended by the manufacturer. For transfections, 0.05 pmol of each reporter pGL3-promoter construct or the external control vector pGL3-Control (Promega) were used per well. In each well, 0.005 pmol thymidine kinase driven *Renilla *luciferase vector pRL-TK (Promega) served as an internal standard for transfection efficiency. For triple-transfection experiments, wells were transfected with 0.1 μg of reporter constructs and 0.01 μg of pRL-TK together with increasing amounts of Sp1 or Sp3 expression plasmid. Luciferase activities were normalized for transfection efficiency according to Renilla activities. For testing effects of Sp-protein overexpression on *PDPN *transcription, MG63 and Saos-2 cells were transfected in 12-wells with 0.6 μg pCMV-Sp1 or pMCS-Sp3, or with 0.3 μg of each vector. SL-2 *Drosophila *cells were transiently transfected in 12-wells with 0.25 μg of pPac vectors together with 0.25 μg of luciferase reporter using FuGene6 (Roche) according to the manufacturer's recommendations. After 48 h, cells were lysed in 1 × passive lysis buffer and luciferase activities were measured by the Dual Luciferase Reporter Assay System (Promega) per the manufacturer's instructions. When using single Luciferase signals, activities were normalized to protein amounts of the lysates as determined by BCA assay (Pierce). Measurements were made using a Lumat LB9507 luminometer (EG&G Berthold).

### Preparation of nuclear extracts

Nuclear extracts were prepared from MG63 and Saos-2 cells with minor variations of the method of Dignam et al. [64]. Briefly, cells were washed twice with ice-cold phosphat-buffered saline, scraped off in PBS and centrifuged 5 min at 1500 × g at 4°C. Cells were resuspended in buffer A (10 mM HEPES, pH 7.9, 1.5 mM MgCl_2_, 10 mM KCl, 0.5 mM DTT, 0.5 mM PMSF), left on ice for 20 min and were lyzed by passing them ten times through a 22 gauge needle. The nuclei were recovered by centrifugation for 6 min at 4500 × g, washed with buffer A, and the nuclear proteins were extracted with high salt buffer B (20 mM HEPES, pH 7.9, 1.5 mM MgCl_2_, 420 mM NaCl, 0.2 mM EDTA, 25% glycerol, 0.5 mM DTT and 0.5 mM PMSF) on a rotating wheel for 30 min at 4°C. Extracts were centifuged for 15 min at 10000 *g*, and supernatants were stored in aliquots at -78°C until use. Protein concentrations were measured by BCA protein assay (Pierce) giving typical protein yields of 3 – 4 μg/μl.

### DNaseI footprint analysis

Footprinting experiments were performed with the Core Footprinting System Kit (Promega). DNA probes were dephosphorylated, labeled at both ends with (γ^32^P)-ATP (Hartmann Analytic) by T4 polynucleotide kinase, and digested with an restriction enzyme that released one of the labeled ends. Probes were purified on an agarose gel and DNA was recovered with the Qiaquick Gel Extraction Kit (Qiagen). 5 ng of the labeled probe were incubated with 5 μg nuclear extract in 50 μl of 25 mM Tris-HCl (pH 8.0), 50 mM KCl, 6.25 mM MgCl_2_, 1 mM EDTA, 20% glycerol and 1 mM DTT for 20 min on ice and then, 50 μl of a 5 mM CaCl_2_/10 mM MgCl_2 _solution was added. After 1 min at room temperature, various amounts (0.15 – 1.5 units) of RQ1 RNase-free DNase I were added in order to obtain an even DNase I ladder from top to the bottom of the gel, mixed gently, and after 2 min of incubation the reaction was terminated with 90 μl stop solution (200 mM NaCl, 30 mM EDTA, 1% SDS, 100 μg/ml yeast RNA). DNA was extracted with phenol/chloroform/isoamylalcohol 1:1:1, precipitated with ethanol, and the pellet was dissolved in loading solution (1:2 0.1 M NaOH:formamide (v/v), 0.1% cylene cyanol, 0.1% bromphenol blue). After heating the probes 2 min at 95°C and chilling on ice, they were loaded onto a 6% polyacrylamide sequencing gel containing 8 M urea and run in 1 × TBE buffer at 1500 Volt. Gels were dried on a filter paper and exposed to a Kodak Phosphor screen. A sequencing reaction of the respective DNA probe was carried out with the Sequenase Version 2.0 DNA Sequencing Kit (USB Corp.) using (α^35^S)-dATP (New England Nuclear) in the reaction mixture and was run side by side with the footprint assay. Images were analyzed with a STORM 830 facility (Molecular dynamics).

### Electrophoretic mobility shift assays (EMSAs)

Double-stranded oligonucleotide probes were 5'-endlabeled with (γ^32^P)-ATP and T4 polynucleotide kinase. EMSAs were carried out in a 10 μl reaction containing 3 μg of nuclear extract, 250 ng poly(dI-dC), 2 μl of 5 × binding buffer (50 mM HEPES, pH 7.9, 50 mM KCl, 25 mM MgCl_2_, 2.5 mM EDTA, 40% glycerol, 5 mM DTT, 0.1% NP40, 200 μg/ml BSA) and labeled oligo probe (80 fmoles, 20.000 cpm) for 15 min on ice. Competitive EMSAs were performed under identical conditions by adding the 50 or 100 fold amount of the same unlabeled doublestranded oligo or an unspecific oligo as negative control. For supershift experiments, 1.5 or 1 μl of anti-human Sp1-, or Sp3-specific antibodies (Santa Cruz) or unspecific rabbit IgG were added to the reaction mix and incubated for 20 min on ice. Protein-DNA complexes were resolved on a 4.5% nondenaturing polyacryamide gel with Tris-EDTA buffer (45 mM Tris, 44.5 mM borate, 1 mM EDTA, pH 8.0) at 350 V at 4°C. Gels were dried on filter paper, exposed 1 to 5 days to a Kodak Phosphor screen and scanned with a STORM 830 facility.

### Western blot analysis

Nuclear extracts or total lysates from MG63 and Saos-2 cells were applied to 10% SDS-PAGE and transferred onto nitrocellulose membrane. Western analysis was performed by incubating the membrane with rabbit anti-Sp1, -Sp3, -TFIIB, or -GAPDH antibodies (Santa Cruz), followed by HRP-conjugated secondary antibodies. Signals were detected by enhanced chemiluminescence using an ECL kit (Amersham Pharmacia). Densitometric quantification was perfomed with the LumiAnalyst 3.0 program (Roche).

### ChIP assay

ChIP assays were performed with a ChIP kit (Upstate) according to the manufacturer's recommendations. For each chromatin immunoprecipitation assay, 2 – 3 × 10^7 ^cells (80 – 90% confluency) were crosslinked with 1% formaldehyde at room temperature for 20 min. Cells were washed twice with ice-cold PBS, scraped from the culture dish and resuspended in SDS lysis buffer (Upstate). Chromatin was fragmented by sonication (Labsonic U, B. Braun) in 20 sec pulses on ice to an average size of 600 bp. The lysates were diluted 1:10 with dilution buffer and pre-cleared by adding 75 μl protein A agarose for 30 min at 4°C. 40 μl of the lysate were removed as control input DNA and microcentrifuged at full speed for 10 sec to pellet residual cell debris. IP was performed by adding 4 μg anti-Sp1 antibody (Upstate), 4 μg anti-Sp3 antibody (Upstate) or 5 μg unspecific rabbit IgG overnight at 4°C under rotation, and an aliquot was incubated without antibody. Immunocomplexes were precipitated by addition of protein A agarose for 1 hr at 4°C. Precipitates were washed once with low salt buffer, once with high salt buffer, once with LiCl buffer and twice with TE buffer (Upstate). Protein/DNA complexes were eluted from the antibodies with 1% SDS, 0.1 M NaHCO_3_, and DNA-protein interactions were reversed by addition of 5 M NaCl and heating to 65°C for 4 hrs. Proteins were digested with proteinase K for 1 hr at 45°C. Remaining DNA was purified by phenol/chloroform extraction and subsequent ethanol precipitation. The DNA was finally resuspended in 40 μl 1 × TE, pH 8.0, and 4 μl of the DNA was used for each PCR reaction. Site-specific PCR was carried out using primer pairs located at binding sites Sp1.1/Sp1.2 (193 bp), Sp1.3 (184 bp) and Sp1.4 (115 bp). PCR primers were designed as 19–26 mers with ~55% GC content. PCR was performed with a hot start, followed by 30 – 35 cycles of 30 s at 95°C, 1 min annealing at 63°C, 65°C and 68°C for each Sp1 site, respectively, and 30 s extension at 72°C. Besides that, input DNA was used concentrated (4 μl) and in 1:4 and 1:16 dilutions in PCR reactions. PCR products were run on a 2% agarose gel and visualized by ethidium bromide staining. Each ChIP experiment was carried out at least three times with similar results.

### Genomic DNA southern blot analysis

Genomic DNA was extracted from MG63 and Saos-2 cells according to Sambrook et al. [65]. Briefly, genomic DNA was isolated by cell lysis with proteinase K (Promega) digestion and extraction with phenol/chloroform. After precipitation, DNA was digested with the restriction enzyme *Pst*I to generate a 3989 bp podoplanin promoter fragment ranging from bp -2799 to bp +1190 and then with the isoschizomeric restriction enzymes *Hpa*II and *Msp*I (NEB) with the binding site 5'-CCGG-3' to release methylation-sensitive and -insensitive fragments. DNA fragments were separated on a 1.5% agarose gel, transferred onto a nylon membrane and hybridized with five radioactively labeled probes (P/B, F2/K, F5/Bs, Ex1, Int1). After hybridization and subsequent washing, autoradiographs were analyzed with a STORM 830 facility.

### Sodium bisulfite genomic DNA sequencing

Bisulfite genomic sequencing was performed as described previously [29]. Briefly, genomic DNA (5 μg) was denatured in 0.3 M NaOH at 37°C for 15 min. Then, 3 M sodium bisulfite (Sigma) and 10 mM hydroquinone (Sigma) were added and samples were incubated at 50°C for 16 h. The modified DNA was purified through the Wizard Clean-Up system (Promega) and denatured by addition of 0.3 M NaOH at 37°C for 15 min. DNA was precipitated, resuspended in 1 mM Tris-HCl, pH 8 and used for PCR reactions. Three regions were amplified with primer pairs specific for bisulfite-reacted upper strands (Table 1). PCR was performed with DNA isolated from four independent bisulfite treatment experiments and PCR products were cloned into pCR2.1-TOPO vector (Invitrogen). Fifty to eighty clones from each reaction were analyzed by sequencing.

### 5-AzaCdR and TSA treatment

Viability of MG63 and Saos-2 cells under the influence of DNA methyltransferase inhibitor 5-azaCdR was tested up to concentrations of 1, 10 and 50 μM, and for histone deacetylase inhibitor TSA up to concentrations of 1, 2 and 3 μM over a period of 72 hrs. 5-azaCdR was added for 48 h at a final concentration of 1 μM, and TSA was added for 24 h alone, or for additional 24 h at the end of 5-azaCdR treatment final concentrations of 0.3 and 0.6 μM. Cells then were used for RNA isolation and subsequent quantitative Northern blotting analysis as described above. Densitometric quantification was perfomed with the LumiAnalyst 3.0 program (Roche).

### In vitro methylation of oligos and reporter plasmids

*Sss*I methylase (NEB) was used for the methylation of EMSA oligos and of *PDPN *promoter-luciferase constructs. Oligos, pGL3-II and pGL3-I plasmid DNA were incubated with methylase according to the manufacturers' recommendations. Region-specific methylation was carried out after excision with *Sac*I/*Xho*I and *Sac*I/*Apa*I to isolate the promoter fragments -1885/+171, -1885/-189 from pGL3-II and -857/+171 and -857/-189 from pGL3-I, as described previously [31]. In each case, half of the DNA was methylated in the absence of S-adenosyl-methionine as mock methylation. The efficiency of methylation was determined with the methylation-sensitive enzyme *Hpa*II. Methylated and mock-methylated fragments were religated into their descendant plasmids and equal amounts were transfected into MG63 and Saos-2 cells.

### Statistical analyses

Statistical relevant differences were assigned to a p-value of at least ≤0.05 using Student's paired t-test.

## List of abbreviations

*PDPN*, gene name for podoplanin; HEPES, 4-(2-hydroxyethyl)-1-piperazineethanesulfonic acid; DTT, dithiothreitol; EMSA, electrophoretic mobility shift assay; 5-azaCdR, 5-aza-2'-deoxycytidine; TSA, trichostatin; S.D., standard deviation.

## Authors' contributions

BH is responsible for the experimental design of this study, has performed the studies shown in Figs 1A; 2C; 5D; 6A,C,D; 9B,C, and has written the manuscript. RK carried out the experiments shown in Figs 1B,C,D,E; 3B; 4; 5A,B,C; 6B; 7; 8 and 9A. CP prepared some of the promoter luciferase constructs. SK performed computer analyses and participated intellectually in the experiments shown in Fig. 9B and 9C. DK coordinated and reviewed the manuscript. All authors have approved the manuscript.
